# Lutein Protects against Methotrexate-Induced and Reactive Oxygen Species-Mediated Apoptotic Cell Injury of IEC-6 Cells

**DOI:** 10.1371/journal.pone.0072553

**Published:** 2013-09-06

**Authors:** Chi-Jen Chang, Ji-Fan Lin, Hsun-Hsien Chang, Gon-Ann Lee, Chi-Feng Hung

**Affiliations:** 1 Division of Pediatric Surgery, Department of Surgery, Shin-Kong Wu Ho-Su Memorial Hospital, Taipei, Taiwan; 2 School of Medicine, Fu-Jen Catholic University, New Taipei, Taiwan; 3 Central Laboratory, Shin-Kong Wu Ho-Su Memorial Hospital, Taipei, Taiwan; 4 Children's Hospital Informatics Program, Harvard-Massachusetts Institute of Technology Division of Health Sciences and Technology, Harvard Medical School, Boston, Massachusetts, United States of America; 5 Department of Chemistry, Fu-Jen Catholic University, New Taipei, Taiwan; University of Kentucky, United States of America

## Abstract

**Purpose:**

High-dose chemotherapy using methotrexate (MTX) frequently induces side effects such as mucositis that leads to intestinal damage and diarrhea. Several natural compounds have been demonstrated of their effectiveness in protecting intestinal epithelial cells from these adverse effects. In this paper, we investigated the protection mechanism of lutein against MTX-induced damage in IEC-6 cells originating from the rat jejunum crypt.

**Methods:**

The cell viability, induced-apoptosis, reactive oxygen species (ROS) generation, and mitochondrial membrane potential in IEC-6 cells under MTX treatment were examined in the presence or absence of lutein. Expression level of *Bcl2, Bad* and ROS scavenging enzymes (including *SOD, catalase* and *Prdx1*) were detected by quantitative RT-PCR.

**Results:**

The cell viability of IEC-6 cells exposed to MTX was decreased in a dose- and time-dependent manner. MTX induces mitochondrial membrane potential loss, ROS generation and caspase 3 activation in IEC-6 cells. The cytotoxicity of MTX was reduced in IEC-6 cells by the 24 h pre-treatment of lutein. We found that pre-treatment of lutein significantly reduces MTX-induced ROS and apoptosis. The expression of SOD was up-regulated by the pre-treatment of lutein in the MTX-treated IEC-6 cells. These results indicated that lutein can protect IEC-6 cells from the chemo-drugs induced damage through increasing ROS scavenging ability.

**Conclusion:**

The MTX-induced apoptosis of IEC-6 cells was shown to be repressed by the pre-treatment of lutein, which may represent a promising adjunct to conventional chemotherapy for preventing intestinal damages.

## Introduction

Cancers are leading causes of mortality in both adults and children. Multiple drugs have been developed to treat cancers. An antifolate, methotrexate (MTX) is widely used to treat cancers and a number of other malignant and nonmalignant diseases. The effect of MTX is attributed to its inhibition of dihydrofolate reductase, purine synthesis or blocking DNA repair, but can cause an acute injury to the intestinal epithelium characterized by reduced mitosis in the crypts and shortened villi [1], [2]. High-dose MTX treatment is often accompanied by adverse effects, such as severe enterocolitis, weight loss, anorexia, diarrhea and intestinal atrophy [3], [4]. MTX induces damage to the villi of the small intestine, leading to decreased surface area of the small intestine. Shortened villi of the small intestine change the physical structure of brush-border membranes and compromise the components of the small intestinal mucosa such as proteins and lipids, and. The chemical and morphological changes in the small intestinal may be triggered by crypt cells injury. In addition to the morphological changes, MTX is known to be a source to decrease the intestinal absorption of nutrients and drugs [5], [6]. MTX is also known as a pro-oxidant compound that causes depletion of the dihydrofolate pool. It directly affects the synthesis of thymidilate, suppresses DNA synthesis, inhibits epithelial proliferation, and induces apoptosis in the small intestinal crypts [7]. Therefore, MTX is widely used for cancer and also for rapidly-dividing cells of the intestinal crypt. Nevertheless, MTX raises an important complication for patients who are undergoing cancer chemotherapy. The clinical application of this drug is limited by its toxic dose-related side effects. Methotrexate has been shown to decrease viability and to induce apoptosis in the small intestine in rats [8]. In order to minimize the side effects in patients taking MTX as chemotherapeutic agent, it is important to reduce the damages and to stimulate tissue repair [9]. Growth factors such as insulin-like growth factor 1 and keratinocyte growth factor were reported to protect mice from gastrointestinal injury by stimulating growth of the damaged intestine [10], [11]. Other studies using vitamin A [6], [12], aged garlic extract (AGE) [8], apricot, beta-carotene [13], melatonin [14] and prostaglandins [15], showed protective effect on the MTX-induced damages to the small intestine in rats.

Lutein is one of the most commonly found carotenoids in deep-yellow vegetables and fruits, including cooked spinach, lettuce, Broccoli, peas, lima beans, orange juice, celery, string beans, and squash [16]. The structure of lutein is similar to that of beta-carotene. Lutein derivatives have various biologic properties such antioxidant, anti-inflammatory and anti-tumor potential. Furthermore, lutein has neuroprotective effects in the retina mainly through its antioxidant ability [17]. Studies also demonstrated that lutein prevents the ischemia reperfusion injury induced by free radical species in the rat small intestine [18]. Apricot and beta-carotene that are structurally similar to lutein also protect the impairment of oxidative stress in the small intestine of rats from MTX-induced damages [13]. However, lutein was reported to suppress cancer cell growth by inducing apoptosis [19], [20]. Thus, it has been suggested that lutein has different effects on tumor cells and normal intestinal cells.

IEC-6 is an immortalized epithelial cell line derived from neonatal rat ileum and has been extensively used as an *in vitro* intestinal model for the research of floate and its transport derivatives [21]. In the present study, we examined the effects of lutein to protect the intestine from MTX-induced cytotoxic injuries in an IEC-6 intestinal epithelial cell chemotherapy damage model.

## Materials and Methods

### Materials

MTX was purchased from Sigma (St. Louis, MO, USA). Stock solution of MTX was prepared in 0.1 M NaOH at 1 mg/ml and diluted 1:10 in 0.1 M phosphate buffered saline (PBS) before the use; the pH of the solution was adjusted to 7.4. Lutein was purchased from ChromaDex Inc. (Irvine, CA, USA) and was dissolved in dimethyl sulfoxide (DMSO). Reagents for cell culture, including Dulbecco's modified Eagle's medium (DMEM), penicillin and streptomycin antibiotic mixture, glutamax, sodium pyruvate and fetal bovine serum were from Invitrogen (Carlsbad, CA, USA). The tetrazolium salt WST-1 (4-[3-(4-Iodophenyl)-2-[4-nitrophenyl]-2H-5-tetrazolio]-1,3-benzene disulfonate) was purchased from Roche Diagnostics (Roche Applied Science, Penzberg, Germany). JC-1 (5,5′,6,6′-tetrachloro-1,1′3,3′-tetraethylbenzimidazolylcarbocyanine iodide), PI (propidium iodide), and H_2_DCFDA (6-carboxy-2′,7′-dichlorodihydrofluorescein diacetate), were purchased from Sigma. Caspase 3 substrate, (Z-DEVD)_2_-R110 was purchased from Bachem (Torrance, CA, USA). Other chemicals unless otherwise stated were obtained from Sigma.

### Cell culture

IEC-6 cells (ATCC#CRL1592) were obtained from American Type Culture Collection (Rockville, MD). Cells were cultured in DMEM supplemented with 10% fetal bovine serum (FBS; Invitrogen), 2 mM GlutaMAX-I (Invitrogen), 100 units/ml penicillin, and 100 μg/ml streptomycin (Invitrogen). Cells were incubated at 37°C in 5% CO_2_. The culture medium was changed every 2 days.

### Cell viability assay

The effects of MTX, alone or in combination with lutein, in cell viability were determined by WST-1 reagent, according to the manufacturer's recommendations. In brief, 4×10^3^ cells were seeded on a 96-well plate and allowed to attach for 24 h. the cells were then treated with MTX, lutein, or MTX in the present of lutein. After the indicated time period, cells were incubated for 1 h with 10 μl of WST-1 reagent and the absorbance was measured using a microplate reader (PowerWave ×340, Bio-Tek Instruments, Inc., Winooski, USA) at 450 nm. In the pretreatment experiment, cells were pretreated 2 h or 24 h with lutein prior to MTX exposure and measured of cell viability.

### Apoptosis assay

Apoptosis induction by MTX was assessed by (a) activation of caspase 3 activity; (b) detection of caspase 3 cleavage by Western blot.

#### 1. Caspase 3/7 activity

Activation of caspase-3 was assayed using (Z-DEVD)_2_-R110 substrate as described [22]. Briefly, cells were plated in 96-well plates and allowed to attach by overnight incubation. The cells were then treated with DMSO (control) or the desired concentrations of MTX in the presence or absence of lutein for 24 h. Subsequently, the cells were directly lyzed by adding caspase 3 assay buffer containing (Z-DEVD)_2_-R110 substrates and incubated at 37°C for 1 hour. The fluorescent intensity of proteolytically released fluorophore R110 was then measured using a plate reader (Victor X2; PerkinElmer, Waltham, MA, USA) with an excitation of 485 nm and emission of 535 nm.

#### 2. Detection of caspase 3 cleavage by Western blot

Cells subjected to the indicated treatment were harvested and lysed, and the protein concentration was determined by a BCA protein assay (Pierce, Rockford, IL, USA). Protein samples were separated by 14% sodium dodecylsulfate polyacrylamide gel electrophoresis (SDS-PAGE) and transferred to polyvinylidene difluoride (PVDF) membranes (Millipore, Billerica, MA, USA) before probing with antibodies (caspase-3 (active), Epitomics, Burlingame, CA; β-actin (as internal control), Sigma). Subsequent immunoblotting procedures were performed using a chemiluminescence procedure (Millipore) per the manufacturer's instructions. The intensity of immunoreactive bands was determined using GeneTools software (Syngene, Frederick, MD, USA) after scanning the developed films. Results are expressed as mean ± standard deviation (S.D.) of three independent experiments.

### Detection of mitochondrial membrane potential

Changes in the mitochondrial membrane potential in cells were measured by flow cytometry using JC-1 as described [23]. Thirty minutes prior to cytometric analysis, JC-1 is added to the cells to a final concentration of 10 μM. Cells are then examined on a FL-1 (530 nm) versus FL-2 (585 nm) dot plot on a FACSCalibur flow cytometer (Becton Dickinson, BD.; NJ. USA). JC-1 has dual emission depending on the state of the mitochondrial membrane potential. It forms aggregates in cells with a high FL-2 fluorescence indicating a normal mitochondrial membrane potential. Loss of the mitochondrial membrane potential results in a reduction in FL-2 and with a concurrent gain in FL-1 fluorescence as the dye shifts from an aggregate to monomeric state. Therefore, the retention of the dye in the cells can be monitored through the increase in FL-1 fluorescence.

### ROS assay

Intracellular ROS generation in cells treated with MTX was measured by flow cytometry following staining with H_2_DCFDA as described [24]. Briefly, 2×10^5^ cells were plated in 6-well culture plates, allowed to attach overnight, and exposed to DMSO (control) or the desired concentration of MTX for the specific time intervals. The cells were stained with 10 μM H_2_DCFDA, and the fluorescence was measured using a FACSCalibur flow cytometer (BD), and the data were analyzed with CellQuest pro software (BD). In some experiments, cells were pretreated for 2 h or 24 h with Lutein prior to MTX exposure and analysis of ROS generation.

### Quantitative RT-PCR

The expression levels of *Bcl-2, Bad*, superoxidase dismutase 1(*SOD*), catalase (*CAT*) and Peroxiredoxin 1 (*Prdx1)* were measured in MTX-treated cells with or without the pretreatment of lutein. Total RNA was isolated using TRIzol reagent (Invitrogen). RNA yields were measured using Nanodrop 2000 (Thermo Scientific, Rockford, IL, USA). First strand cDNA was synthesized from total RNA (2 μg) by First Strand cDNA Synthesis Kit (Thermo Scientific) using oligo dT primers. Q-PCR was performed in a 10 μl reaction that contained 0.5 μl of the cDNA and 1× KAPA SYBR® FAST Universal qPCR Master Mix (Kapa biosystems, Woburn, MA, USA), using the following PCR parameters: 95°C for 3 min followed by 40 cycles of 95°C for 10 s, 60°C for 30 s followed by melt curve: 65°C to 95°C increment 0.5°C for 5 s. The expression level of GAPDH was used as internal control. The relative expression was calculated using the comparative Ct and expressed as fold of control. The specific primer pairs used to amplify genes are listed in Table S1.

### Statistical analysis

All experiments were performed in triplicate, and results are presented as the mean ± S.D. Student's *t*-test was employed to compare control and experimental parameters with *P*≤0.05 considered as statistically significant. SigmaPlot (vers. 10) was utilized for data analysis and graphical presentation.

## Results

### Dose- and time-dependent cytotoxicity in IEC-6 cells upon MTX treatment

**Figure 1:**
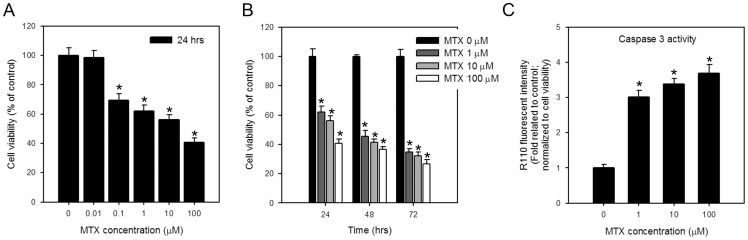
MTX induces dose- and time-dependent cell viability lost and activates caspase 3 activities in IEC-6 cells. IEC-6 cells were treated with (A) different concentration of MTX (0, 0.01, 0.1, 1, 10 and 100 μM) for 24 hrs, or (B) 0, 1, 10, 100 μM of MTX for 24, 48, and 72 hrs, and the cell viabilities were determined by WST-1 assay. (C) The caspase 3 activities were detected in cells treated with indicated concentration of MTX for 24 hrs. Values are means ± S.D. of at least three independent experiments. *, *p*<0.05.

Most studies described a dose- and time-dependent effect of MTX on the inhibition of cell proliferation and the induction of apoptosis [25], [26], [27]. After exposing IEC-6 cells to different concentrations of MTX for 24 hours, we observed a clear effect made by the dose. As shown in Figure 1A, the cell viability was reduced to 56.1±3.3 % and 40.7±2.9 % in cells treated with 10 and 100 μM MTX, respectively. MTX treatment also exhibited a time-dependent reduction of cell viability in IEC-6 cells. When treating cells with 10 μM of MTX, the cell viability at 24, 48 and 72 h was reduced to 56.1±3.3, 41.5±2.1 and 32.3±2.5 %, respectively (Figure 1 B). To test whether the reduced cell viability is associated with the induction of apoptosis in MTX treated cells, we detected the caspase 3 activity in cells treated with different concentrations of MTX at 24 h. The result showed a dose-dependent activation of caspase 3 (Figure 1C), indicating induction of apoptosis and subsequently leading to cell death.

### Induction of ROS and loss of mitochondrial membrane potential in IEC-6 cells treated with MTX

**Figure 2:**
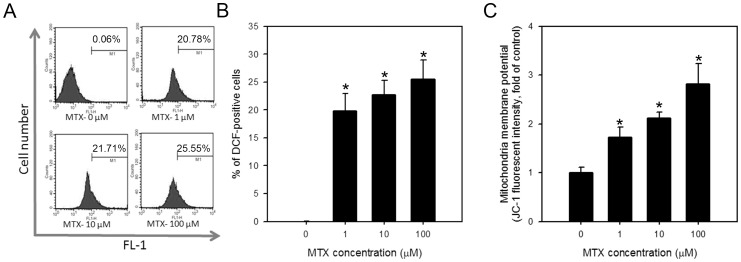
Generation of ROS and disruption of mitochondrial membrane potential in MTX-treated IEC-6 cells. (A and B) Generation of ROS and (C) loss of mitochondrial membrane potential were examined by H_2_DCFDA and JC-1 dye, respectively, through flow cytometry after 24 hrs in cells treated with indicated concentration of MTX. Data were obtained from 10,000 events and presented as the percentage of cells positive for JC-1 monomer or DCF (green fluorescent). The representative histograms showing increment of FL-1 positive cells were given in (A). The values are shown as the mean ± S.D. of three independent experiments. *, *p*<0.05.

MTX was reported to induce ROS in the intestinal mucosa in rats [28]. We therefore detected the induction of ROS in IEC-6 cells treated with different dose of MTX. As shown in Figure 2A and B, the ROS-positive cells were increased to 19.8±3.1, 22.7±2.6 and 25.6±3.4 % in cells treated with 1, 10 and 100 μM MTX compared to control. Because mitochondria are considered as the main source of internal ROS in the cells, we further detected the changes of mitochondrial membrane potential in IEC-6 cells treated with MTX. A dose-dependent loss of mitochondrial membrane potential was observed as the fluorescent intensity of monomeric JC-1 increased to 1.7±0.2, 2.1±0.1 and 2.8±0.4 fold in IEC-6 cells treated with 1, 10 and 100 μM MTX, respectively, compared to control (Figure 2C). These results indicate that MTX induces the loss of mitochondrial membrane potential and generation of ROS in IEC-6 cells.

### The effect of lutein on IEC-6 cell viability and caspase 3 activation

**Figure 3:**
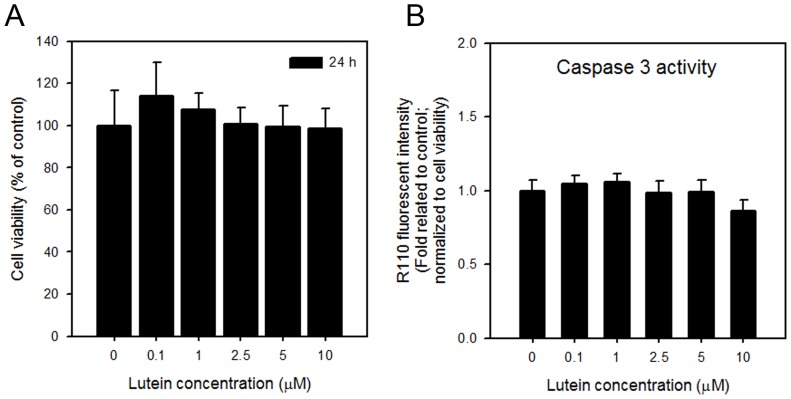
The effect of lutein on IEC-6 cells. (A) Cell viability and (B) caspase 3 activities of IEC-6 cells treated with 0, 0.1, 1, 2.5, 5 and 10 μM lutein for 24 hrs were determined. Data are shown as mean ± S.D. of quadruplicate measurements of each condition that were repeated three times.*, *p*<0.05.

Lutein is one of the naturally occurring carotenoids and has been proved to reduce H_2_O_2_ induced ROS stress in cells [29], [30], [31]. Therefore, we hypothesized that lutein could suppress MTX-induced ROS and thus protect the damage of MTX in IEC-6 cells. To test this theory, we first investigated the effect of lutein on the viability of IEC-6 cells. As shown in Figure 3A, administration of lutein raging from 0.1 to 10 μM showed no effect on the viability of IEC-6 cells. And as expected, the caspase 3 activity was not altered by the administration of lutein in IEC-6 cells (Figure 3B).

### Pretreatment of lutein inhibits MTX-induced cell viability lost and apoptosis

**Figure 4:**
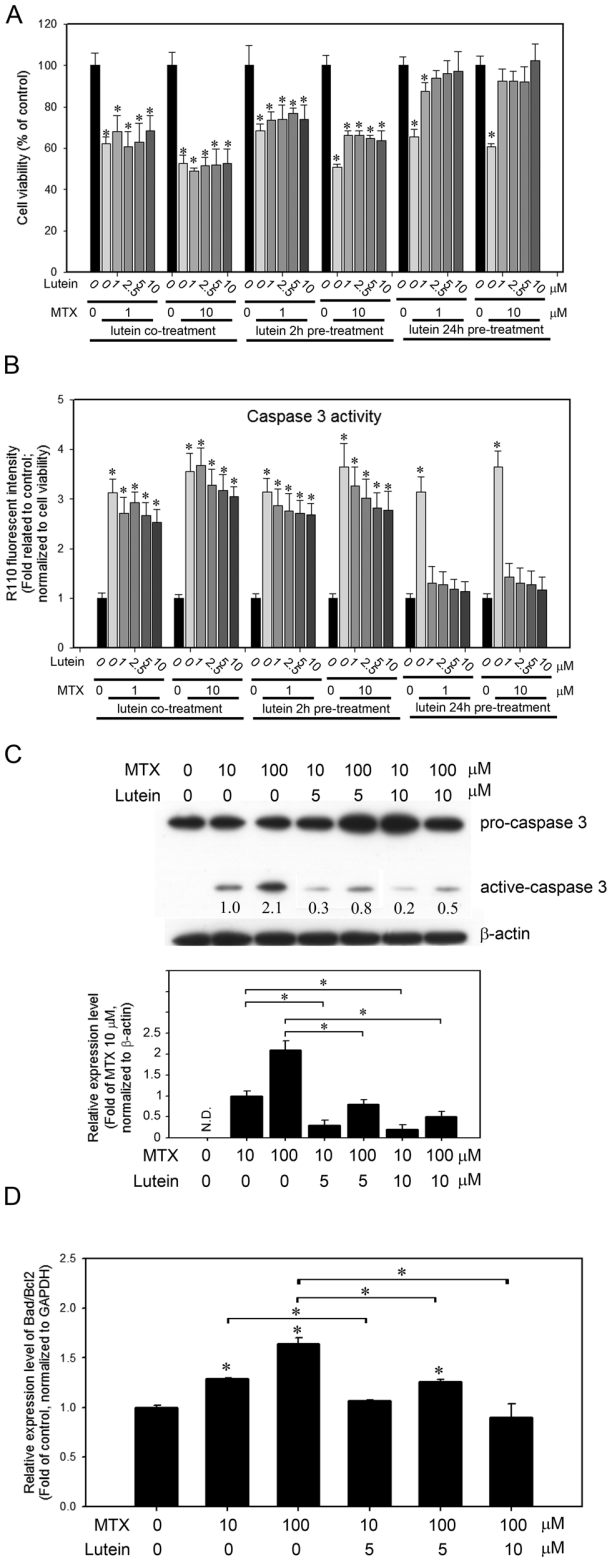
The impact of lutein on MTX-treated IEC-6 cells. Cells with co-treatment of MTX and lutein, or pretreated with lutein for 2 or 24 hrs were measured for their (A) cell viability and (B) caspase 3 activity. (C) The cleaved caspase 3 (active-caspase 3) in MTX-treated cells with or without lutein pretreatment for 24 hrs was detected by Western blot. The relative expression level of active-caspase 3 was quantitated by denstometric scanning and is presented as the fold of the cells treated with 10 μM of MTX. *, *p*<0.05. (D) The relative expression level of *Bad/Bcl2* in MTX-treated cells with or without lutein pretreatment for 24 hrs was measured by quantitative RT-PCR. Data shown represent the means of quadruplicate measurements of each condition and were repeated three times. The results are presented as fold of untreated control (means ± S.D.); *, *P*<0.05.

To test whether lutein inhibits MTX-induced cell injury, we first detected the effects of lutein in cells treated with MTX. The cells were pretreated with lutein for 2 or 24 h prior to the administration of MTX, or co-treated with lutein and MTX. The cell viability was then detected. As shown in Figure 4A, cell viability was reduced to 62.1±3.3 and 52.6±4.1 % in cells treated with 1 or 10 μM of MTX. Co-treatment of 1, 2.5, 5 or 10 μM of lutein had no effect on the viability lost in MTX-treated cells. Two hours of lutein pre-treatment has little but no significant effect in cells treated with 1 μM of MTX. Cell viability was slightly increased in 10 μM MTX treated cells that pretreated with lutein for 2 h. The rescue of cell viability lost was observed in cells pre-treated with lutein for 24 h then treated with 1 or 10 μM MTX. We next detected the activity of caspase 3 in MTX-treated cells with the lutein co-treatment or pre-treatment. The results were consistent with the cell viability, cells pre-treated with lutein for 24 h suppressed the MTX-induced activation of caspase 3 (Figure 4B). These results were further confirmed by direct monitoring the expression levels of cleavage caspase 3 in MTX-treated cells with 24 h lutein pre-treatment using Western blot (Figure 4C). The apoptotic index (expression ratio of *Bad/Bcl2*) also showed decreased apoptosis in MTX-treated cells with 24 h pre-treatment of lutein (Figure 4D). The data suggests that lutein may protect cells against MTX-induced apoptosis.

### Lutein suppresses MTX-induced ROS generation

**Figure 5:**
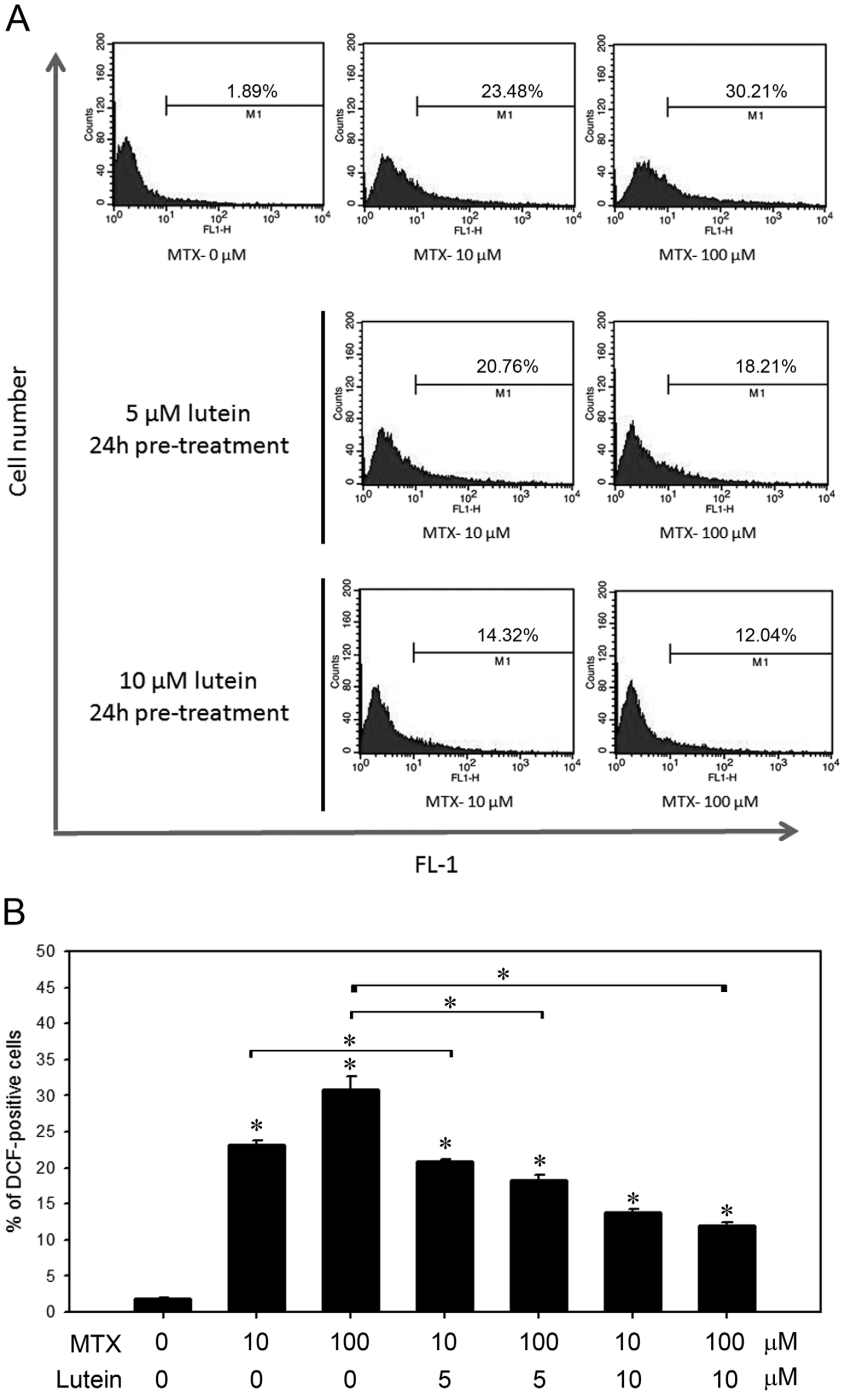
Pre-treatment of lutein suppresses MTX-induced ROS generation in IEC-6 cells. MTX-treated cells with or without the pre-treatment of lutein for 24 hrs were measured for ROS generation. (A) The representative histograms of each condition. (B) The percentage of DCF-positive cells. Data shown were from the means of triplicate measurements of each condition and were repeated three times. *, *P*<0.05.

We next evaluated the anti-oxidant activity of lutein in cells treated with MTX. We hypothesized that lutein suppresses MTX-induced ROS generation to inhibit apoptosis induction that causes lost cell viability. As shown in Figure 5, ROS generation in cells treated with MTX was suppressed by the pre-treatment of lutein. Pre-treatment of 5 and 10 μM of lutein suppressed the percentage of DCF-positive cells from 30.8±1.83% to 18.2±0.79% and 12.0±0.41%, respectively, in 100 μM MTX-treated IEC-6 cells.

### Expression of ROS scavenging enzymes in MTX-treated IEC-6 cells with or without lutein pretreatment

**Figure 6:**
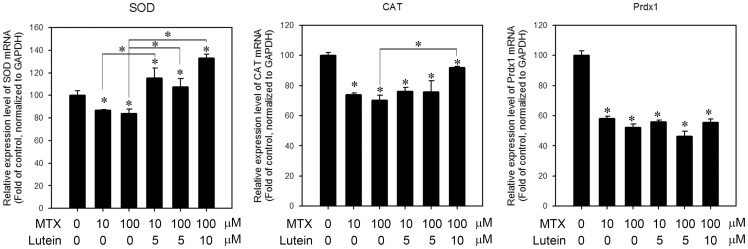
The expression level of ROS scavenging enzymes in MTX-treated cells with or without lutein pre-treatment. The expression level of (A) SOD, (B) catalase and (C) Prdx1 was measured in cells treated with 10 and 100 μM MTX with or without 5 or 10 μM of lutein pre-treatment for 24 h. Data shown represent the means of quadruplicate measurements of each condition and were repeated three times. The results are presented as percentage of untreated control (means ± S.D.); *, *P*<0.05.

To gain insight of the ability of lutein on suppressing ROS generation induced by MTX, we monitored the expression of ROS scavenging enzymes, including *SOD, CAT* and *Prdx1*. The expressions of these anti-oxidant enzymes were all decreased upon MTX treatment (Figure 6). The expression of *SOD* was increased in cells pre-treated with lutein for 24 h. The expression level of *SOD* was even higher in lutein treated cells than the control cells. The expression of *CAT* was also up-regulated in cells pre-treated with lutein, but was lower than control. The expression of *Prdx1* was reduced in MTX-treated cells and was not elevated with the pre-treatment of lutein.

## Discussion

In the present study, we have shown that MTX treatment caused IEC-6 cell death in a time- and dose-dependent manner via apoptosis, as demonstrated by the loss of IEC-6 cells viability, the induced activation of caspase-3 activity and the increased cleavage caspase 3. The MTX-induced loss of viable IEC-6 cells was prevented by pre-treatment with lutein. Our data suggests that lutein suppresses MTX-induced ROS generation by activating the expression of ROS scavenging enzymes and therefore reduces MTX-induced apoptosis in IEC-6 cells. These results suggested that lutein may be useful for alleviating the adverse effects generated by the cancer chemotherapy with MTX.

Caspases are crucial mediators of programmed cell death. Among them, caspase-3 is a frequently activated death protease, catalyzing the specific cleavage of many key cellular proteins. Caspase-3 activity in IEC-6 cells exposed to MTX was significantly up-regulated. Aged garlic extract (5 μM) was shown to significantly reduce activation of caspase-3 induced by MTX [26]. Caspase-3, a key enzyme for execution of apoptosis in many instances, also plays a central role for MTX-induced apoptosis in IEC-6 cells.

The intrinsic apoptotic pathway is characterized by permeabilisation of the mitochondria and by release of cytochrome c into the cytoplasm. Cytochrome c then forms a multi-protein complex known as the ‘apoptosome’ and initiates activation of the caspase cascade through caspase 9. Active caspase 9 cleaves and activates downstream caspases-3, -6, and -8, leading to apoptosis [32]. Mitochondrial permeability is also related to the increased generation of reactive oxygen species (ROS), which plays a role in the degradation phase of apoptosis [33]. In our study, the MTX-treated IEC-6 cells were shown increased release of ROS with disrupted mitochondrial membrane potential and active caspase-3 that leads to apoptosis.

Recent reports demonstrated that MTX induces ROS generation and causes significant reduction in the antioxidant enzyme levels, sensitizing the cells to ROS [18], [28], [34], [35], [36], [37]. Oxidative stress was reported to contribute to MTX-induced small intestinal toxicity in rats [38]. Administration of N-acetylcysteine (NAC) decreases the MTX-induced damage to the small intestine in rats [39]. Oncologists contended that antioxidants interfere with radiation and some chemotherapeutic treatments because those modalities kill by generating free radicals that are neutralized by antioxidants and that folic acid interferes with MTX. However, a previous study reviewing fifty human clinical randomized or observational trials involving 8,521 patients shown non-prescription antioxidants and other nutrients do not interfere with therapeutic modalities for cancer. Furthermore, they enhance the therapeutic outcomes by decreasing side effects and protect normal tissue [40]. We hereby showed that the pre-treatment of lutein suppressed the ROS induced by MTX and increased the expression of SOD and catalase in the intestinal epithelial cells. SOD and catalase are ROS scavenging enzymes, and have been demonstrated to protect small intestinal damages induced by ischemia reperfusion [41]. Absorption of lutein in human intestinal epithelium was demonstrated to be associated with the scavenger receptor class B member 1 (SR-BI) protein [42]. Furthermore, the bioaccessibility of lutein in small intestine is greater than β-carotene or lycopene [43]. Our results indicate that pre-treatment of lutein inhibited MTX-induced ROS generation and apoptosis in IEC-6 cells, and suggest that lutein may have a potential role in adjuvant cancer chemotherapy by reducing the intestinal damage of anti-cancer drugs.

## Supporting Information

Table S1 **QPCR primers used in this study.**
(DOC)
