# Supplementary material for: Lutein Protects against Methotrexate-Induced and Reactive Oxygen Species-Mediated Apoptotic Cell Injury of IEC-6 Cells
Source: PLoS One. 2013 Sep 6;8(9):e72553. doi: 10.1371/journal.pone.0072553 (PMC3765170; doi:10.1371/journal.pone.0072553)
Supplement: Table S1 — QPCR primers used in this study. (DOC) [file pone.0072553.s001.doc]

Article title: Lutein protects against methotrexate-induced and reactive oxygen species-mediated apoptotic cell injury of IEC-6 cells.

Authors: Chi-Jen Chang, Ji-Fan Lin, Hsun-Hsien Chang, Gon-Ann Lee, and Chi-Feng Hung.

Supplementary Table S1.

Table S1. QPCR primers used in this study.

| Gene | GenBank Acc. no. | Forward primer | Reverse primer |
| --- | --- | --- | --- |
|  |  | 5’🡪3’ | |
| GAPDH | NM_017008 | TGACTCTACCCACGGCAAGTT | TGATGGGTTTCCCGTTGATGA |
| Bcl-2 | NM_016993 | CCGGGAGAACAGGGTATGATAA | CCCACTCGTAGCCCCTCTG |
| Bad | NM_022698 | TCCGAAGAATGAGCGATGA | ACTGGATAATGCGCGTCCA |
| SOD | NM_017050 | CGAGCATGGGTTCCATGTC | CTGGACCGCCATGTTTCTTAG |
| CAT | NM_012520 | ACAACTCCCAGAAGCCTAAGAATG | GCTTTTCCCTTGGCAGCTATG |
| Prdx1 | NM_057114 | GTGATTGGAGCTTCTGTGGA | CAAGGGAATGTTCATGGGTC |
